# 再生障碍性贫血诊断与治疗中国指南（2022年版）

**DOI:** 10.3760/cma.j.issn.0253-2727.2022.11.001

**Published:** 2022-11

**Authors:** 中华医学会血液学分会红细胞疾病（贫血）学组

中华医学会血液学分会红细胞疾病（贫血）学组在广泛征集专家建议和意见的基础上，结合再生障碍性贫血（AA）最新诊治进展及我国国情对2017年版AA专家共识进行更新，制订《再生障碍性贫血诊断与治疗中国指南（2022年版）》。

一、AA概述及发病机制

AA是一种骨髓造血衰竭（BMF）综合征。其年发病率在我国为0.74/10万，可发生于各年龄组，AA高发年龄分别为15～25岁的青壮年和65～69岁的老年人[1]，男、女发病率无明显差异。AA分为先天性及获得性。目前认为T淋巴细胞异常活化、功能亢进造成骨髓损伤在原发性获得性AA发病机制中占主要地位，新近研究显示辅助性T细胞亚群Th1/Th2分化偏移[2]、调节性T细胞（Treg）及NK细胞调节功能不足[3]–[5]、Th17[6]、树突状细胞（DC细胞）[7]以及巨噬细胞[8]等功能异常甚至某些遗传背景都参与了AA发病。先天性AA较为罕见，主要为范可尼贫血（FA）、先天性角化不良（DKC）、先天性纯红细胞再生障碍（DBA）、Shwachmann-Diamond综合征（SDS）等。绝大多数AA属获得性，本指南主要讨论原发获得性AA。

二、AA的诊断建议

（一）诊断AA的实验室检测项目

1. 推荐检测项目：①血常规检查：白细胞计数及分类、红细胞计数及形态、HGB水平、网织红细胞百分比和绝对值、血小板计数和形态。②不同平面多部位骨髓穿刺：至少包括髂骨和胸骨。骨髓涂片分析：造血细胞增生程度；粒、红、淋巴系细胞形态和各阶段百分比；巨核细胞数目和形态；小粒造血细胞面积；是否有异常细胞等。③骨髓活检：至少取2 cm骨髓组织（髂骨）标本用以评估骨髓增生程度、各系细胞比例、造血组织分布（有无灶性CD34^+^细胞分布等）情况，以及是否存在骨髓浸润、骨髓纤维化等。④流式细胞术检测骨髓CD34^+^细胞数量、阵发性睡眠性血红蛋白尿症（PNH）克隆（CD55、CD59、Flaer）。⑤肝、肾、甲状腺功能，病毒学［包括肝炎病毒、EB病毒（EBV）、巨细胞病毒（CMV）、细小病毒B19等］及免疫球蛋白、补体、免疫固定电泳检查。⑥血清铁蛋白、叶酸和维生素B_12_水平。⑦免疫相关指标检测：T细胞亚群（如CD4^+^、CD8^+^、Th1、Th2、Treg等）、细胞因子（如IFN-γ、IL-4、IL-10等）、NK细胞亚群、自身抗体和风湿抗体，大颗粒淋巴细胞白血病相关标志检测。⑧细胞遗传学：常规染色体核型分析、荧光原位杂交［del（7）、del（7q−）、+8、del（5q）、del（20q）等］以及遗传性疾病筛查（儿童或有家族史者推荐做染色体断裂试验），胎儿血红蛋白检测。⑨其他：心电图、腹部超声、超声心动图及其他影像学检查（如胸部X线或CT等）等。

2. 建议检测项目：有条件的医院可开展以下项目：①骨髓造血细胞膜自身抗体检测；②端粒长度及端粒酶活性检测、端粒酶基因突变检测；③二代测序（NGS）检测有无先天性骨髓衰竭性疾病相关基因突变及克隆造血分子标志。

（二）AA诊断标准

1. 血常规检查：全血细胞（包括网织红细胞）减少，淋巴细胞比例增高。至少符合以下三项中两项：HGB<100 g/L；PLT<50×10^9^/L；中性粒细胞绝对值（ANC）<1.5×10^9^/L。

2. 骨髓穿刺：多部位（不同平面）骨髓增生减低或重度减低；小粒空虚，非造血细胞（淋巴细胞、网状细胞、浆细胞、肥大细胞等）比例增高；巨核细胞明显减少或缺如；红系、粒系细胞均明显减少。

3. 骨髓活检（髂骨）：全切片增生减低，造血组织减少，非造血细胞增多，网硬蛋白不增加，无异常细胞。

4. 除外检查：必须除外先天性（表1）和其他获得性、继发性BMF（表2）。

**表1:** 与再生障碍性贫血相鉴别的先天性全血细胞减少症

疾病	临床特征
先天性无巨核细胞性血小板减少症	常染色体隐性遗传，TPO受体c-Mpl基因突变所致；血小板减少或全血细胞减少；骨髓衰竭
先天性角化不良症	遗传方式有X连锁遗传、常染色体显性或隐性遗传；皮肤色素异常、口腔白斑、指甲营养不良三联征；全血细胞减少，肺纤维化等
范可尼贫血	大部分属常染色体隐性遗传，少数（FANCB亚型）为X连锁遗传；主要表现为血细胞减少、先天畸形、幼年癌症，染色体断裂试验阳性，易进展为MDS/AML
RUNX1种系突变	血小板减少或全血少，易进展为MDS/AML
GATA-2缺失综合征	反复感染（分枝杆菌、病毒、真菌等），淋巴水肿，疣，肺泡蛋白沉积症，易进展为MDS/AML
SAMD9/9L异常	MIRAGE（骨髓增生异常，感染，生长受限，肾上腺发育不全，生殖异常，肠病），易进展为MDS/AML
重症先天性中性粒细胞减少症	中性粒细胞减少，反复细菌感染
先天性中性粒细胞减少伴胰腺功能不全综合征	常染色体隐性遗传，多数患者有SBDS基因突变，表现为骨髓衰竭，胰腺外分泌功能不全，易进展为MDS/AML

注 MDS：骨髓增生异常综合征；AML：急性髓系白血病

**表2:** 与AA相鉴别的其他获得性或继发全血细胞减少症

疾病或临床表现	鉴别要点
PNH相关（AA/PNH）	依据疾病及PNH向AA转化的阶段不同，患者的临床表现不同。检测外周血红细胞和白细胞表面GPI锚链蛋白可以鉴别
低增生性MDS/AML	低增生性MDS具备如下特点：增生减低，一系或多系病态造血；外周血可见幼稚细胞；骨髓活检可见网状纤维、CD34^+^细胞增加以及前体细胞异常定位（ALIP）
自身抗体介导的全血细胞减少	包括Evans综合征等。可检测到外周成熟血细胞的自身抗体或骨髓未成熟血细胞的自身抗体，患者可有全血细胞减少并骨髓增生减低，但外周血网织红细胞或中性粒细胞比例往往不低甚或偏高，骨髓红系细胞比例不低且易见“红系造血岛”，Th1/Th2降低（Th2细胞比例增高）、CD5^+^ B细胞比例增高，血清IL-4和IL-10水平增高，对糖皮质激素和（或）大剂量静脉滴注丙种球蛋白、CD20单抗、CTX等治疗反应较好
大颗粒淋巴细胞（LGL）白血病	可表现为全血细胞减少，和（或）脾大及B症状等。流式细胞术检测外周血持续性LGL数量增多，TCR基因重排等检测证实LGL为克隆性增殖
霍奇金淋巴瘤或非霍奇金淋巴瘤	可表现为全血细胞减少、骨髓增生减低、骨髓涂片可见局部淋巴瘤细胞浸润。AA患者淋巴细胞显著增高，但系正常淋巴细胞，可通过免疫分型和基因重排检测与淋巴瘤细胞进行区分。其他如脾肿大等特征也可作为鉴别AA与淋巴瘤的依据
原发性骨髓纤维化	可表现为全血细胞减少，外周血可检测到泪滴样异常红细胞、幼稚粒细胞/幼稚红细胞，脾肿大。骨髓易干抽，骨髓活检可见巨核细胞增生和异型巨核细胞，网状纤维和（或）胶原纤维
分枝杆菌感染	有时表现为全血细胞减少和骨髓增生减低，可见肉芽肿、纤维化、骨髓坏死和噬血征象。结核分枝杆菌一般没有特征性肉芽肿。抗酸杆菌属于不典型分枝杆菌感染，其常被泡沫样巨噬细胞吞噬。如果考虑结核，应进行骨髓抗酸染色和培养
神经性厌食或长期饥饿	可表现为全血细胞减少、骨髓增生减低、脂肪细胞和造血细胞丢失，骨髓涂片背景物质增多，HE染色为浅粉色，吉姆萨染色亦可观察到
原发免疫性血小板减少（ITP）	部分AA患者初期仅表现为血小板减少，后期出现全血细胞减少，需与ITP相鉴别。这类AA患者骨髓增生减低、巨核细胞减少或消失。这种表现在ITP中并不常见。可用于鉴别早期AA及ITP
MonoMac综合征	骨髓增生减低同时外周血单核细胞减低或极度减低可能提示该诊断

注 AA：再生障碍性贫血；PNH：阵发性睡眠性血红蛋白尿症；GPI：糖基磷脂酰基醇；MDS：骨髓增生异常综合征；AML：急性髓系白血病；MonoMac综合征：分枝杆菌易感的单核细胞缺乏综合征

（三）AA严重程度确定（Camitta标准）

1. 重型AA（SAA）诊断标准：（1）骨髓细胞增生程度<正常的25％；如≥正常的25％但<50％，则残存的造血细胞应<30％。（2）血常规需具备下列三项中的两项：ANC<0.5×10^9^/L；网织红细胞绝对值<20×10^9^/L；PLT<20×10^9^/L。（3）若ANC<0.2×10^9^/L，则诊断为极重型AA（VSAA）。

2. 非重型AA（NSAA）诊断标准：未达到SAA。根据是否依赖血制品输注，将NSAA分为输血依赖型（TD-NSAA）和非输血依赖型（NTD-NSAA），TD-NSAA有向SAA转化风险[9]。成分输血指征：HGB≤60 g/L；PLT≤10×10^9^/L，或PLT≤20×10^9^/L伴有明显出血倾向。平均每8周至少1次成分输血且输血依赖持续时间≥4个月者称为TD-NSAA[10]。

（四）AA鉴别诊断

AA应与其他引起全血细胞减少的疾病相鉴别，见表2。AA属于BMF。BMF可以分为先天性和获得性两种，而获得性BMF又分为原发性和继发性。

1. 其他原发性BMF：其他原发性BMF主要包括：（1）源于造血干细胞质量异常的BMF，包括PNH、骨髓增生异常综合征（MDS）和意义未明克隆性血细胞减少（CCUS）等；（2）自身抗体介导的BMF；（3）意义未明的血细胞减少（ICUS），是某特定疾病的前期阶段，可发展为MDS或其他血液病，也可能是尚未认知的疾病。

2. 继发性BMF：造成继发性BMF的因素较多，主要包括：（1）造血系统肿瘤，如毛细胞白血病（HCL）、大颗粒淋巴细胞白血病（LGL）、多发性骨髓瘤（MM）等；（2）其他系统肿瘤浸润骨髓；（3）骨髓纤维化；（4）严重营养性贫血；（5）急性造血功能停滞；（6）肿瘤性疾病因放化疗所致骨髓抑制等。

三、AA的治疗建议

（一）AA的治疗原则

SAA一经确诊应尽早启动本病治疗，研究显示SAA诊断30 d内启动治疗疗效明显优于30 d后启动治疗组[11]。确诊为SAA患者及TD-NSAA的标准疗法：对年龄≤40岁且有HLA相合同胞供者的SAA患者，如无活动性感染和出血，首选HLA相合同胞供者造血干细胞移植（MSD-HSCT）[12]。对无HLA相合同胞供者和年龄>40岁的患者首选免疫抑制治疗（IST）［抗胸腺/淋巴细胞球蛋白（ATG/ALG）+环孢素A（CsA）］联合促血小板生成素受体激动剂（TPO-RA）和（或）其他促造血的治疗方案[13]–[15]；HLA相合无关供者造血干细胞移植（MUD-HSCT）或单倍体造血干细胞移植（Haplo-HSCT）目前提倡适用于IST无效的年轻SAA患者。对NTD-NSAA可采用CsA联合TPO-RA和（或）其他促造血治疗（图1）。

**图1:**
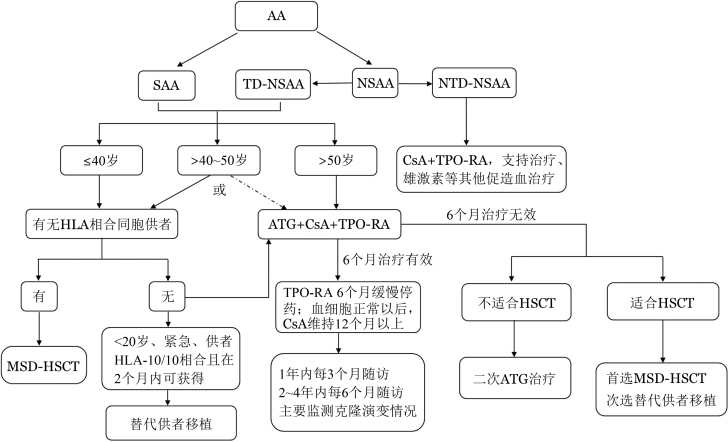
再生障碍性贫血（AA）治疗路线图 注 SAA：重型再生障碍性贫血；NSAA：非重型再生障碍性贫血；TD-NSAA：输血依赖非重型再生障碍性贫血；NTD-NSAA：非输血依赖非重型再生障碍性贫血；HLA：人类白细胞抗原；ATG：抗胸腺细胞球蛋白；CsA：环孢素A；TPO-RA：促血小板生成素受体激动剂；HSCT：造血干细胞移植；MSD-HSCT：同胞全相合HSCT

在一线治疗选择时，除了关注年龄、是否存在合并症、疾病的严重程度、造血干细胞移植合并症指数评分（HCT-CI）外，还需评价是否存在影响预后的其他因素。如存在多个IST预后良好因素（表3），则更倾向于一线使用IST联合TPO-RA治疗；如存在多项IST预后不良因素，如端粒显著缩短、不良基因突变（ASXL1、TP53、RUNX1、DNMT3A）、合并难以控制的活动性感染、从NSAA逐渐发展到SAA等，若条件允许尽量选择HSCT，TPO-RA联合IST选择需权衡利弊。无论如何，在TPO-RA联合IST疗效明显提升背景下，选择MUD-HSCT或Haplo-HSCT时，应充分衡量患者风险与获益[17]。

**表3:** 再生障碍性贫血（AA）免疫抑制治疗（IST）疗效良好的预测因素

预测因素
1. 年龄小
2. 病情较轻
3. 网织红细胞绝对值>25×10^9^/L且淋巴细胞绝对值>1.0×10^9^/L
4. 染色体异常+8或del（13q）
5. 存在PIGA基因突变或阵发性睡眠性血红蛋白尿克隆
6. 端粒长度不能预测血液学反应，但长端粒组IST后总生存率高
7. BCOR和BCORL1突变[16]

（二）支持疗法

1. 成分血输注：红细胞输注指征一般为HGB<60 g/L。老年（≥60岁）、代偿反应能力低（如伴有心、肺疾病）、需氧量增加（如感染、发热、疼痛等）、氧气供应缺乏加重（如失血、肺炎等）时红细胞输注指征可放宽为HGB≤80 g/L，尽量输注悬浮红细胞。

存在血小板消耗危险因素（感染、出血、使用抗生素或ATG/ALG等）者或SAA预防性血小板输注指征为PLT<20×10^9^/L，病情稳定者为PLT<10×10^9^/L。发生严重出血者则不受上述标准限制，应积极输注单采浓缩血小板悬液。重症感染（如败血症患者）或ATG治疗期间，尽量维持PLT≥20×10^9^/L。因产生抗血小板抗体而导致无效输注者应输注HLA配型相合的血小板。

粒细胞缺乏伴不能控制的细菌和真菌感染，广谱抗生素及抗真菌药物治疗无效可以考虑粒细胞输注治疗，建议连续输注3 d以上。治疗过程中预防及密切注意粒细胞输注相关不良反应，如输血相关性急性肺损伤、同种异体免疫反应及发热反应。

2. 其他保护措施：SAA患者应予保护性隔离，有条件者应入住层流病房；避免出血，防止外伤及剧烈活动；必要的心理护理。

3. 感染的预防和治疗：口腔护理及高压无菌饮食，必要时可预防性应用抗真菌药物。欲进行移植及ATG/ALG治疗者建议预防性应用抗细菌、抗病毒及抗真菌治疗。造血干细胞移植（HSCT）后需预防卡氏肺孢子菌感染，如用复方磺胺甲唑（SMZco）。由于已有一些报道提示接种疫苗可诱发BMF或AA复发，除非绝对必要否则不主张接种疫苗。AA患者发热同样遵循“中性粒细胞减少伴发热”的治疗原则处理。

4. 祛铁治疗：长期反复输血超过20 U和（或）血清铁蛋白水平高于1 000 µg/L的患者，有条件可进行肝脏、心脏MRI检查，明确铁过载程度。根据血细胞数量和脏器功能情况酌情祛铁治疗，以铁螯合剂为主，推荐应用去铁胺、地拉罗司。近期研究显示艾曲泊帕具有一定的祛铁作用，疗效与铁螯合剂相当，因此在IST联合TPO-RA治疗时代，祛铁治疗的策略是否面临改变尚需研究证实[18]。

（三）AA本病治疗

1. IST（ATG/ALG+CsA）联合促造血治疗：经NIH和RACE两个临床研究证实，已将IST联合TPO-RA方案确立为不适合移植SAA患者的一线治疗方案。

（1）ATG/ALG：目前ATG/ALG使用无年龄上限，对于>60岁SAA患者需评估合并症以及患者一般情况是否适合应用。兔源ATG（法国）剂量为2.5～3.5 mg·kg^−1^·d^−1^，猪源ALG（中国）剂量为20～30 mg·kg^−1^·d^−1^，连续使用5 d。输注之前均应按照相应药品制剂说明进行皮试和（或）静脉试验，试验阴性方可接受ATG/ALG治疗。每日用ATG/ALG时同步应用肾上腺糖皮质激素防止过敏反应。急性期不良反应包括：超敏反应、发热、僵直、皮疹、高血压或低血压及液体潴留。血清病反应（关节痛、肌痛、皮疹、轻度蛋白尿和血小板减少）一般出现在ATG/ALG治疗后1周左右，因此糖皮质激素应足量用至15 d，随后减量，一般2周后减完（总疗程4周），出现血清病反应者则静脉应用肾上腺糖皮质激素冲击治疗。

第1次ATG/ALG治疗无效或复发患者可选择HSCT或第2次ATG/ALG治疗。选择第2次ATG/ALG治疗，应与前次治疗间隔3～6个月，第2个疗程的ATG/ALG，宜尽可能采用动物种属来源与前次不同的ATG/ALG剂型，以减少过敏反应和严重血清病发生的风险。

（2）CsA：CsA联合ATG/ALG用于SAA时，CsA口服剂量为3～5 mg·kg^−1^·d^−1^，建议与ATG/ALG同时应用。CsA治疗AA的确切有效血药浓度并不明确，有效血药浓度窗较大，一般目标血药浓度（C0谷浓度）为成人150～250 µg/L，儿童酌减。临床可根据血药浓度及疗效调整CsA的应用剂量。CsA减量过早会增加复发风险，IST联合TPO-RA治疗SAA的方案中，CsA足量应用6个月或疗效达平台期后建议持续用药12～24个月后停药[13]。CsA主要的不良反应为消化道反应、齿龈增生、色素沉着、肌肉震颤、肝肾功能损害，少数出现头痛和血压变化；因此，服用CsA期间应定期检测血压、肝肾功能，出现上述不良反应可通过CsA减量或停药予以纠正。

（3）TPO-RA：TPO-RA包括海曲泊帕、艾曲泊帕、阿伐曲泊帕、罗米司亭等，其中海曲泊帕在我国获批难治成人SAA适应证，艾曲泊帕在美国获批治疗初诊及难治SAA，其他TPO-RA的临床研究均正在进行，目前多为探索性治疗。

艾曲泊帕在ATG应用第1天同时给药可获得最佳疗效，起始剂量为75 mg/d，根据疗效情况可每两周增加25 mg/d进行剂量爬坡，最大剂量为150 mg/d。血小板正常后缓慢减药，不要骤停，尤其对于老年及未达CR的患者[13],[19]。海曲泊帕治疗难治成人SAA，推荐起始剂量7.5 mg/d，每2周加量2.5 mg/d，最大剂量15 mg/d[20]。艾曲泊帕及海曲泊帕均应空腹服用，避免与抗酸药物或含多价金属阳离子的食物，如奶制品等同服，或当有合并用药时根据药品说明书调整药物剂量。

艾曲泊帕及海曲泊帕最常见的不良反应为肝脏毒性，在治疗过程中应严密监测肝功能变化，目前没有证据表明艾曲泊帕增加克隆造血（CH）的发生率，但CH出现更早，需定期监测[14]。

对艾曲泊帕或海曲泊帕联合IST治疗SAA无效患者，可尝试TPO-RA之间的转换，如罗米司亭用于治疗艾曲泊帕无效的AA患者，起始剂量每周20 µg/kg，70％患者在3个月出现不同程度血液学反应[21]。但仍需更多临床研究数据指导用药选择。

2. 其他方案：

（1）其他免疫抑制剂：也有研究显示抗CD52单抗、他克莫司、雷帕霉素、环磷酰胺（Cy）等对于难治、复发SAA有效。

（2）其他促造血治疗：雄激素可以刺激骨髓红系造血，减轻女性AA患者月经期出血过多，且具有端粒调节作用，常用的雄激素包括：司坦唑醇、十一酸睾酮、达那唑等。国内研究显示重组人血小板生成素（TPO）及白细胞介素11（IL-11）联合IST也可治疗SAA[22]。G-CSF在IST治疗时代具有加速中性粒细胞恢复、协助控制感染等作用，到目前为止无证据表明G-CSF可增加克隆演变的风险。也有研究显示加用促红细胞生成素（EPO）可加速造血恢复[23]。

3. 随访：接受IST联合TPO-RA方案治疗的SAA患者应定期随访，以便及时评价疗效和不良反应。主要监测指标包括：①造血功能：血常规（带有网织红细胞绝对值）、骨髓检测（骨髓增生程度、形态学、流式细胞术检测细胞分群变化）；②免疫指标：T、B、NK及DC细胞各亚群数量及功能；③克隆演变：染色体、FISH、PNH克隆，MDS/急性髓系白血病（AML）二代测序、端粒长度；④用药不良反应：肝肾功能、电解质、血糖等。建议随访观察点为ATG/ALG用药后3个月、6个月、9个月、1年、1.5年、2年、2.5年、3年、3.5年、4年、5年、10年。

4. IST疗效的影响因素：在IST时代，IST治疗有效的预测因素见表3。TPO-RA的加入，提升了网织红细胞绝对值在（10～30）×10^9^/L的SAA患者的疗效，使这部分患者疗效与网织红细胞绝对值>30×10^9^/L的SAA患者疗效相当。目前研究显示IST联合TPO-RA疗效的影响因素仍然限于年龄及残存骨髓造血指标[24]–[25]。

（四）HSCT

MSD-HSCT目前仍被认为是SAA与TD-NSAA适合移植患者的首选治疗方案。对IST无效、适合移植但无HLA相合同胞供者的SAA与TD-NSAA患者，也可采用替代供者移植，包括：MUD-HSCT、Haplo-HSCT和脐血移植（CB-HSCT）。

1. MSD-HSCT：

（1）适用条件：年龄≤40岁、有HLA相合同胞供者的SAA与TD-NSAA患者；年龄超过40岁的SAA患者，在IST治疗失败后，条件允许也可采用MSD-HSCT。有经验的移植中心将MSD-HSCT一线治疗年龄放宽至≤50岁，IST治疗失败挽救治疗年龄放宽至50～60岁[26]–[27]。

（2）移植预处理和移植物抗宿主病（GVHD）预防：以大剂量Cy（200 mg·kg^−1^·d^−1^）和兔源ATG（10 mg·kg^−1^·d^−1^）作为预处理方案，使用甲氨蝶呤联合CsA预防GVHD。对于植入失败率相对较高的患者类型如≥40岁、病程长、重度输血患者，则给予强化预处理方案，在Cy-ATG基础上增加氟达拉滨（Flu）或白消安（BU）或小剂量全身放疗（TBI）以保证植入[28]。

（3）疗效及预后：研究显示以Flu/Cy/ATG（Flu 120 mg·m^−2^·d^−1^+Cy 120～200 mg·kg^−1^·d^−1^+ATG 10 mg·kg^−1^·d^−1^）或Cy/ATG（Cy 200 mg·kg^−1^·d^−1^+ATG 10 mg·kg^−1^·d^−1^）方案作为MSD预处理方案，5年生存率最高，可达91％。多因素相关分析显示影响MSD生存的预后因素为：受体年龄、是否使用含ATG的预处理方案[29]。

2. MUD-HSCT：多用于IST失败后的二线治疗，在一些特殊条件下，如预计IST疗效不佳或<20岁的SAA或VSAA患者，骨髓库能找到HLA-10/10全合无关供者，情况紧急且在2个月之内能实施HSCT的，可将MUD-HSCT作为一线治疗[30]。MUD-HSCT治疗SAA常以Flu/Cy/ATG（Flu 120 mg·m^−2^·d^−1^+Cy 120～200 mg·kg^−1^·d^−1^+ATG 10 mg·kg^−1^·d^−1^）±低剂量TBI为预处理方案和甲氨蝶呤联合CsA预防GVHD。

3. Haplo-HSCT：Haplo-HSCT可作为缺乏MSD或MUD的补充治疗选择[31]–[33]，在有经验的移植中心Haplo-HSCT可用于缺乏MSD患者的一线治疗。

Haplo-HSCT多以BU/Cy/ATG（Bu 6.4 mg·kg^−1^·d^−1^+ Cy 200 mg·kg^−1^·d^−1^+ATG 10 mg·kg^−1^·d^−1^）为预处理方案，以甲氨蝶呤联合CsA和吗替麦考酚酯预防GVHD；移植后环磷酰胺（PTCY）体系也在SAA的Haplo-HSCT取得进展[34]。

4. CB-HSCT：使用脐血作为SAA患者无关供者移植物来源的潜在优势是脐血获取容易，可及时进行移植，对HLA不相合具有更好的耐受性，当患者缺乏HLA相合无关供者时可以考虑行脐血移植。对于IST联合TPO-RA治疗失败患者，又缺乏HLA相合供者，只要脐血细胞计数足够（理想情况下总有核细胞>4×10^7^/kg），可作为一个有效的挽救性治疗手段[35]。

5. 其他探索性治疗：间充质干细胞治疗：间充质干细胞是骨髓造血微环境的重要成分，具有促进造血干细胞的分化和增殖、抗炎、修复损伤组织、调节机体免疫等作用，可促进移植造血干细胞的归巢，改善造血微环境，产生多种细胞因子，加速造血功能的恢复，降低GVHD的发生。

（五）特殊情况AA的处理

1. 伴有PNH克隆的AA患者的处理：在AA患者中可检测到少量PNH克隆，患者骨髓细胞减少但并不出现溶血，通常仅单核细胞和中性粒细胞单独受累，并且仅占很小部分。一些证据表明PNH克隆预示着IST反应更好，推荐对这些患者的处理同无PNH克隆的AA患者。但伴有明显PNH克隆（>50％）及伴溶血临床及生化指标的AA患者慎用ATG/ALG治疗，以针对PNH治疗为主。

2. 妊娠AA患者的处理：AA可发生于妊娠过程中，有些患者需要支持治疗。AA患者妊娠后，疾病可能反复或进展，尤其对于未达到完全缓解的患者。对于妊娠AA患者主要是给予支持治疗，输注血小板维持患者PLT≥20×10^9^/L。不推荐妊娠期使用ATG/ALG、HSCT或雄激素，可予CsA治疗。妊娠期间应该严密监测患者孕情、血常规和重要脏器功能。

3. 肝炎相关性AA的处理：肝炎相关性AA大都发生在肝炎发生后的2～3个月内。如果AA发病前有黄疸史（通常为发病前的2～3个月）则提示可能为肝炎相关性AA。肝功能检查有利于发现肝炎相关性AA。肝炎相关性AA的肝炎病原学检查可为阴性。针对肝炎应该检测甲肝抗体、乙肝表面抗原、丙肝抗体及EBV。合并肝炎的AA病情一般较重，对治疗反应差，预后不良。在目前国内可获得的TPO-RA中，阿伐曲泊帕虽然现阶段临床适应证为“择期行诊断性操作或者手术的慢性肝病相关血小板减少症的成年患者”，但对于肝炎相关AA或AA伴有肝功能异常者，可尝试使用阿伐曲泊帕治疗。

4. 老年AA的治疗：IST联合TPO-RA为首选治疗，部分有MSD的患者，条件允许可以考虑HSCT。尽管对于SAA或TD-NSAA患者，ATG联合CsA比单用CsA疗效更好；但是，对于老年患者，ATG治疗相关毒副作用更大、风险更高，因此是否应用仍需谨慎，如应用可酌情减量。研究显示CsA联合TPO-RA一线治疗可使无法接受ATG治疗的AA患者获益。其他治疗包括单药CsA、雄激素及阿仑单抗。不耐受或拒绝IST的患者也可考虑中医中药及支持对症治疗。

四、AA的疗效标准

1. SAA的IST疗效标准：

完全缓解（CR）：HGB>100 g/L；ANC>1.5×10^9^/L；PLT>100×10^9^/L。

部分缓解（PR）：脱离成分血输注，不再符合SAA诊断标准。

无效（NR）：仍满足 SAA诊断标准。

2. NSAA的IST疗效标准：

完全缓解（CR）：同SAA疗效标准。

部分缓解（PR）：脱离成分血输注（若既往输血依赖），或至少一系细胞数目增加两倍或达正常，或任何一系血细胞基线水平上升：HGB>30 g/L（如治疗前<60 g/L）、ANC>0.5×10^9^/L（如治疗前<0.5×10^9^/L）、PLT>20×10^9^/L（如治疗前<20×10^9^/L）。

无效（NR）：疾病进展，或未能达到上述有效指标。
